# Nocturnal Light Pollution Synergistically Impairs Glucose Metabolism With Age and Weight in Monkeys

**DOI:** 10.1155/2024/5112055

**Published:** 2024-12-10

**Authors:** Shuxing Wang, Xuange Cheng, Zihao Liang, Zhenyi Chen, Jiankai Zhang, Qiang Xu

**Affiliations:** ^1^Department of Anatomy, Medical School, Foshan University, Foshan, Guangdong Province, China; ^2^Department of Food and Pharmaceutical Sciences, Qingyuan Polytechnic, Panlong Park, Qingcheng District, Qingyuan City 511510, Guangdong Province, China; ^3^Qingyuan Hospital of Traditional Chinese Medicine, Qingyuan City, China; ^4^Primate Research Center, Institute of Zoology, Guangdong Academy of Sciences, Guangzhou, China; ^5^Department of Anatomy, Guangdong University, Dongguan, China

**Keywords:** diabetes, glucose metabolism, impaired fasting glucose tolerance, insulin, light pollution, melatonin, monkey

## Abstract

Over the past decades, the global prevalence of Type 2 diabetes mellitus (T2D) and impaired glucose tolerance (IGT) has been increasing at an epidemic rate, yet the exact cause remains unknown. It is widely accepted that glucose metabolism can be impaired by circadian rhythms and sleep disturbances. Concurrently, exposures to light at night have been closely linked to circadian and sleep disturbances. However, there is no direct experiment on primates to demonstrate the precise extent of how serious light pollution impairs glucose metabolism, whether people will eventually become accustomed to this environment, and whether the pollution has synergistic impairing effects with aging and weight on glucose metabolism. To quantitatively address these questions, 137 cynomolgus were exposed to three distinct nocturnal light intensities for consecutive 10 months. Monthly glucose metabolism assessments were conducted. Data pertaining to the mortality rate of preexisting diabetes, incidence of light-induced diabetes and IGT, and alterations in insulin secretion were collected and analyzed. The results show that nocturnal light (1) caused premature deaths in individuals with preexisting diabetes; (2) intensity-dependently induced diabetes and IGT in previous healthy monkeys; (3) intensity-dependently reduced melatonin secretion; (4) had a synergistic impairing effect on glucose metabolism with aging and weight; and (5) although monkeys would eventually adapt to the environment, the disrupted glucose metabolism would not fully recover in most individuals. In conclusion, nocturnal light is associated with the global high prevalence of T2D and IGT. The harmful effects of light pollution on glucose metabolism are synergistic with age and weight.

## 1. Introduction

The concentration of normal blood glucose is regulated and maintained within strict levels through various mechanisms. These mechanisms include increasing glucose output when circulating glucose levels drop, decreasing glucose output, and increasing tissue glucose uptake when glucose levels rise [1]. Diabetes mellitus is a chronic, noncommunicable disease that arises when the body is unable to produce sufficient insulin or fails to effectively utilize the available insulin, leading to a common state of hyperglycemia in the affected individual [2, 3]. Diabetes exists in two primary forms: Type 1 and Type 2. Patients with Type 1 diabetes experience a lack of insulin production from the pancreatic *β*-cells. In contrast, those with Type 2 diabetes have normal or even elevated insulin secretion from the *β*-cells compared to healthy individuals. However, their bodies are unable to effectively utilize the insulin, leading to the coexistence of hyperglycemia and hyperinsulinemia within a single individual. Overall, T2D accounts for over 90% of the patients with diabetes and represents a significant global health issue. Furthermore, in addition to those diagnosed with diabetes, a significantly larger population falls into the category of IGT, a condition characterized by the body's diminished capacity to maintain normal blood glucose levels. Without appropriate intervention, this condition may progress to T2D [4]. According to reports, the incidence of T2D and IGT rises in tandem with urbanization, and in urban areas, it is higher than in rural areas, sometimes even twice as high [5].

Normal exposure to light is essential for our daily health [6], whereas environmental light pollution poses harmful effects on both humans and animals [7–9]. Regarding glucose metabolism, studies provide evidence that (1) it is modulated in accordance with circadian rhythms [1, 10–13], (2) both sleep and circadian rhythm play crucial roles in preserving insulin sensitivity [14–18], and (3) it can sometimes be highly responsive to nocturnal light, to the extent that even brief exposure can significantly impair glucose tolerance [19]. However, there still lacks a systematic study on primates to date regarding the extent to which glucose metabolism will be disrupted by nocturnal light, whether the condition will eventually become accustomed to it, and what the period is. Additionally, it remains questionable whether light will act as an impairing factor in synergy with age, weight, and sex. For a considerable period, the field has relied on nonprimate animals as models for obesity and diabetes [20]. However, nonhuman primates, particularly those with a large size and long lifespan, may serve as the most pertinent analogs for humans. The objective of this study is to address the aforementioned questions quantitatively by employing experimental methods in cynomolgus, a species known for its glucose metabolism and physiological and pathophysiological characteristics that closely resemble those of humans.

## 2. Materials and Methods

### 2.1. Animals

A total of 137 cynomolgus monkeys (comprising 127 males and 10 females) aged between 8 and 23 years (with an average of 13.08 years ± 4.42 SD) and weighing between 3.4 and 15.1 kg (with an average of 8.14 kg ± 1.94 SD) were enrolled in the study. The experimental protocol was approved by the Institutional Animal Care and Use Committee at the Guangdong Institute of Zoology, Guangdong Academy of Sciences. All monkeys originated from Guangdong Landau Biotech Inc., an institution accredited by AAALAC and CNSA for its experimental nonhuman primate breeding and research environment. Immediately prior to their introduction into the environment, the monkeys' health was assessed based on their appearance, movement, mood, and general laboratory test results, which indicated that they were in a healthy condition. Monkey groups were subsequently allocated into rooms based on their prior social relationships in a randomized manner. People who allocated the monkey were unaware of the experimental procedure, including the night brightness in the rooms. None of the monkeys' characteristics, such as age, gender, weight, or glucose metabolism status, were predetermined. Throughout the duration of the experiment, the research team and veterinary staff conducted daily checks on the rooms twice to ensure that all monkeys were in good health.

### 2.2. The Living Environment

Nine rooms were utilized, each with dimensions of 15 × 5 × 3.5 m (depth, width, and height, respectively). Each room features a south-facing back window measuring 0.5 × 2.5 m, a top window spanning 3 × 5 m, and a metal net door (5 × 3.5 m) that leads into a 2-m-wide corridor on the northern side. Additionally, there is an ad libitum access food tray (1 × 1 m) on the floor, a push-drink distilled water fountain, six-level shelves for sitting and playing on the wall, and recreational equipment including a swing, tumbling barrel, log, and balls.

### 2.3. Lighting Condition, Background Noise, and Temperature

The experiment was conducted at the Tropic of Cancer (north latitude 23°26⁣′) with the exception of nocturnal brightness. The brightness during the day was determined by direct sunlight filtering through the windows and scattered light. Considering that white light from a lamp and blue light from a screen are the most common pollution types in the dark hours of a modern living environment, the light spectrums and brightness in each room were accordingly provided by light tubes on the ceiling of the corridor, which shone through the net door (Supporting Information 1). Regardless of the time of day, every room had shadowy areas. The controlled lighting environment commenced once baseline data (Month 0) for all monkeys had been gathered. Throughout the duration of the experiment, the lights were illuminated daily from 5 PM to 8 AM.

Background noise was not intentionally restricted. Typically, the living environment was peaceful, and the room temperature was maintained between 18°C and 30°C.

### 2.4. Adjust of Living Condition for Individual Monkeys

During the experiment, the monkeys were typically housed in groups. However, any monkey that was bullied by others (1/137) or exhibited any diabetic complications would be promptly moved to a normal circadian environment. It would be treated by a veterinarian and raised separately in a cage (1.2 × 1.5 × 1.5 m). For statistical purposes, any data already collected from these monkeys remains valid. Diabetic complications included, but were not limited to, coma, skin ulcers (1/137), cataracts (1/137), and other related conditions.

### 2.5. Food, Fruit, and Cookie

Food was not designated as an experimental factor. A standard food pellet was provided twice daily at 9 AM and 3 PM, each lasting for 2 h. Fruits, including apples, bananas, sliced cucumbers, sweet potatoes, carrots, tomatoes, pitaya, and peaches (one kind each time), were provided every other day at 12 PM with unlimited access for a duration of 1 h. Cookies, peanuts, and popcorn were provided irregularly. On days of test and blood sampling (fasting 13–17 h), the meal at 9 AM would be avoided, but the monkeys had unrestricted access to drinking water, fruit, or their next meal once they woke up from anesthesia.

### 2.6. FBG, HbA1c, Insulin, C-Peptide, and Melatonin Concentration Testing

Monkeys were anesthetized monthly by receiving an intravenous injection of 1% sodium pentobarbital (50 mg/kg) to minimize unexpected fluctuations in blood glucose levels, and blood samples were drawn 30 min later. The FBG was measured on-site using the Ascensia Breeze Blood Glucose Monitoring System (Newbury, Berks, United Kingdom). The acceptable range for FBG levels was set at 1.1–33.3 mmol/L. Any concentration found to be below 1.1 was disregarded, while any concentration exceeding 33.3 was recorded as 33.3 for statistical analysis.

The plasma concentrations of HbA1c, insulin, C-peptide, and melatonin were separately evaluated using enzyme-linked immunosorbent assay (ELISA) kits (R&D System, Beijing, China) and analyzed by Huanya Biomedicine Technology Co. Ltd. (Beijing, China). The results were read using a microplate reader (Multiskan MK3, Thermo Scientific, Beijing, China) at a wavelength of 450 nm. Any uncertain HbA1c results, whether they are too high or too low, such as those that cannot be determined due to hemolysis, and results below 2.6%, will be discarded.

### 2.7. Verify of the Diabetes Condition

After an intravenous injection of glucose solution, the diabetes was confirmed in 12 randomly selected monkeys from the light-induced diabetes (LID) group using the 2hGTT (5 g/kg weight). This test was adapted from the IGT test in humans, which measures blood glucose levels after consuming a standardized amount (75 g) of glucose. All 12 monkeys tested positive for diabetes.

### 2.8. Retrospective Grouping of Monkeys According to Glucose Metabolic Status

Hyperglycemia, typically indicated by elevated blood glucose levels as reflected by FBG, is the primary and most frequently utilized marker of diabetes. Depending on the severity of the hyperglycemic condition, patients can be categorized into two groups: those with diabetes and those with impaired fasting glucose tolerance (IFG). The latter is a prediabetic condition characterized by elevated FBG levels but below the diagnostic threshold for diabetes [21]. For the retrospective grouping of monkeys, we utilized the clinical diagnostic criteria for diabetes that are currently employed in humans [21, 22]. However, we also took into account the concentration of HbA1c, a glycosylated hemoglobin that serves as an indicator of the average blood glucose level over a period ranging from weeks to months. Additionally, weight loss was another diagnostic criterion in our study, but it was evaluated in conjunction with FBG.

Based on the collected data, the monkeys were categorized into four groups: (i) SDM (spontaneous diabetes mellitus, consisting of monkeys diagnosed with diabetes at the onset of the formal experimental session), (ii) LID (consisting of monkeys that developed diabetes during the experimental session), (iii) IFG (consisting of monkeys with elevated FBG and/or HbA1c level but below diabetes diagnosis standard), and (iv) NGT (monkeys with normal glucose tolerance, all the remaining monkeys). The diagnosis of the monkeys that died during the experimental session (eight out of 137, three from SDM, two from LID, and three from the NGT group) was based on the data collected prior to their deaths. Notably, a female monkey that was enrolled in the 6-month preliminary session at a normal glucose level developed diabetes at Month 2. She survived for an additional 4 months in the environment and another 6 months of normal circadian period before being reenrolled in the formal experimental session with a diabetic status. This monkey was counted into the LID group in animal number, but all other data collected from it during the formal session was statistically used in the SDM group. Following this adjustment, the diagnostic rates are comprehensively listed in Table 1.

### 2.9. Statistical Analysis

Raw data were collected for gender, age, weight, concentrations of FBG, HbA1c, insulin, C-peptide, and melatonin. All statistical tests in this report were two-tailed. To produce statistical results, Microsoft Excel, GraphPad InStat 3.10, and SigmaPlot 11.0 for Windows were utilized.

Using GraphPad InStat 3.10, an ordinary one-way ANOVA was executed to compare raw data across the entire batch or within a subgroup of monkeys. This was done to determine if there were any significant differences among various testing time points. Subsequently, the Tukey–Kramer multiple comparison test was applied to compare data from each time point against the baseline of the same group. To determine if there were any differences between the two subgroups, raw data were compared using GraphPad InStat 3.10 ordinary one-way ANOVA, followed by the Tukey–Kramer multiple comparison test to compare data from each time point with the other group at the same point, thereby identifying the source of the difference and the significance of its magnitude. The data were presented as mean ± standard error (se). Differences were found to be statistically significant at the *α* = 0.05 level. The *p* value, *F* value, animal numbers (*N*), and degrees of freedom (DF) for the ANOVA results are listed in Supporting Information 2, 3, 4, and 5. The sources of the difference and the significance of the magnitude were displayed in the corresponding figures.

The correlation between the night brightness and concentrations of FBG, HbA1c, insulin, and C-peptide in overall or subgroups of monkeys was calculated in SigmaPlot 11.0 for Windows by running the linear regression, using the median of nocturnal light intensities of 13, 35, and 75 Lm as independent, while concentrations of FBG, HbA1c, insulin, or C-peptide of each monkey lived in the environments or in subgroups of LID, IFG, and NGT were used as dependents. The time course of the correlation coefficient between predicted concentrations of FBG and light, age, weight, light plus age, light plus weight, or light plus age plus weight was calculated by running linear regression or multiple linear regression in SigmaPlot. Median nocturnal light intensity was used as the dependent variable, while baseline age and the average weight of a monkey during the experiment session were used as independent variables.

The concentrations of plasma melatonin in the fifth month were compared using one-way repeated AVOVA in SigmaPlot among different light intensities.

## 3. Results

### 3.1. Light Pollution Intensity-Dependently Inhibited Melatonin Production

**Figure 1:**
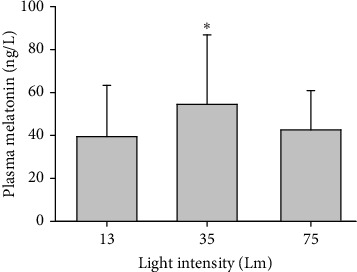
The plasma melatonin concentration in monkeys at Experimental Month 5 showing that at this time point, the melatonin concentration was significantly higher in monkeys exposed to 35 Lm (*N* = 57) nocturnal light blue light compared to those exposed to either 75 Lm white (*N* = 37) or 13 Lm neon light (*N* = 43). ⁣^∗^*p* < 0.05 versus the other two light intensities.

Testing from the Experimental Month 5 sample found that compared to the plasma melatonin in monkeys exposed to the nocturnal brightness of 35 Lm light blue, both that exposed to 75 Lm white and 13 Lm flash neon were statistically lower (Figure 1).

### 3.2. Exposure to Light Pollution Deteriorated Glucose Metabolism in SDM Monkeys

The SDM rate was 5.84% (8/137). In this group, three monkeys died prematurely (37.5%, 3/8). Of these, two monkeys were from the 75 Lm room, including a 17-year-old female who died in the second experimental month with multiple skin ulcers and an 18-year-old male who passed away in the sixth month without any apparent diabetes complications. Another was an 18-year-old male from the 13 Lm flash neon room who died in the sixth month with cataracts in both eyes.

**Figure 2:**
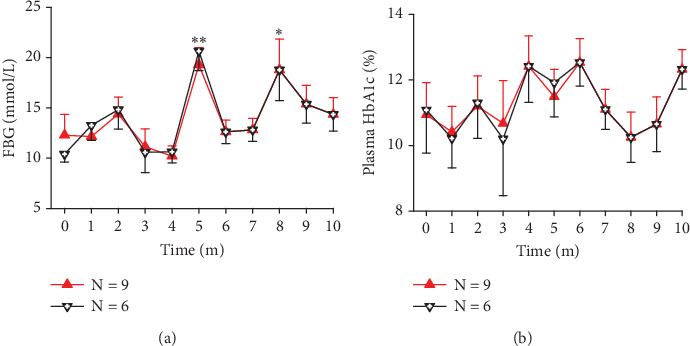
The time course of FBG and HbA1c levels in spontaneous diabetic monkeys showing that exposure to nocturnal light pollution exacerbates glucose metabolism in previously diabetic monkeys. (a) Trend of FBG concentration in SDM monkeys (*N* = 6; ⁣^∗^*p* < 0.05 and ⁣^∗∗^*p* < 0.01 vs. 0). (b) Trend of HbA1c concentration in SDM monkeys (*p* = 0.5897). Time, experimental time in month (m). Error bar, standard error.

In the remaining SDM monkeys, exposure to light pollution led to a decline in glucose metabolism ability, evidenced by elevated average FBG concentrations at later time points (Figure 2(a), *N* = 6, *p* = 0.001 excluding data from the prematurely dead; *N* = 9, *p* = 0.0053 including the dead). Despite the high HbA1c concentrations observed in most SDM monkeys at the onset of the experiment, there was a slight increase in HbA1c levels at one of the later time points for every monkey. However, one-way ANOVA did not reveal statistically significant differences, likely due to the significant interindividual variance at each time point (Figure 2(b)).

### 3.3. Routine and Long-Term Exposure to Light Pollution Intensity-Dependently Induced Diabetes and IFG in Previously Healthy Monkeys

**Figure 3:**
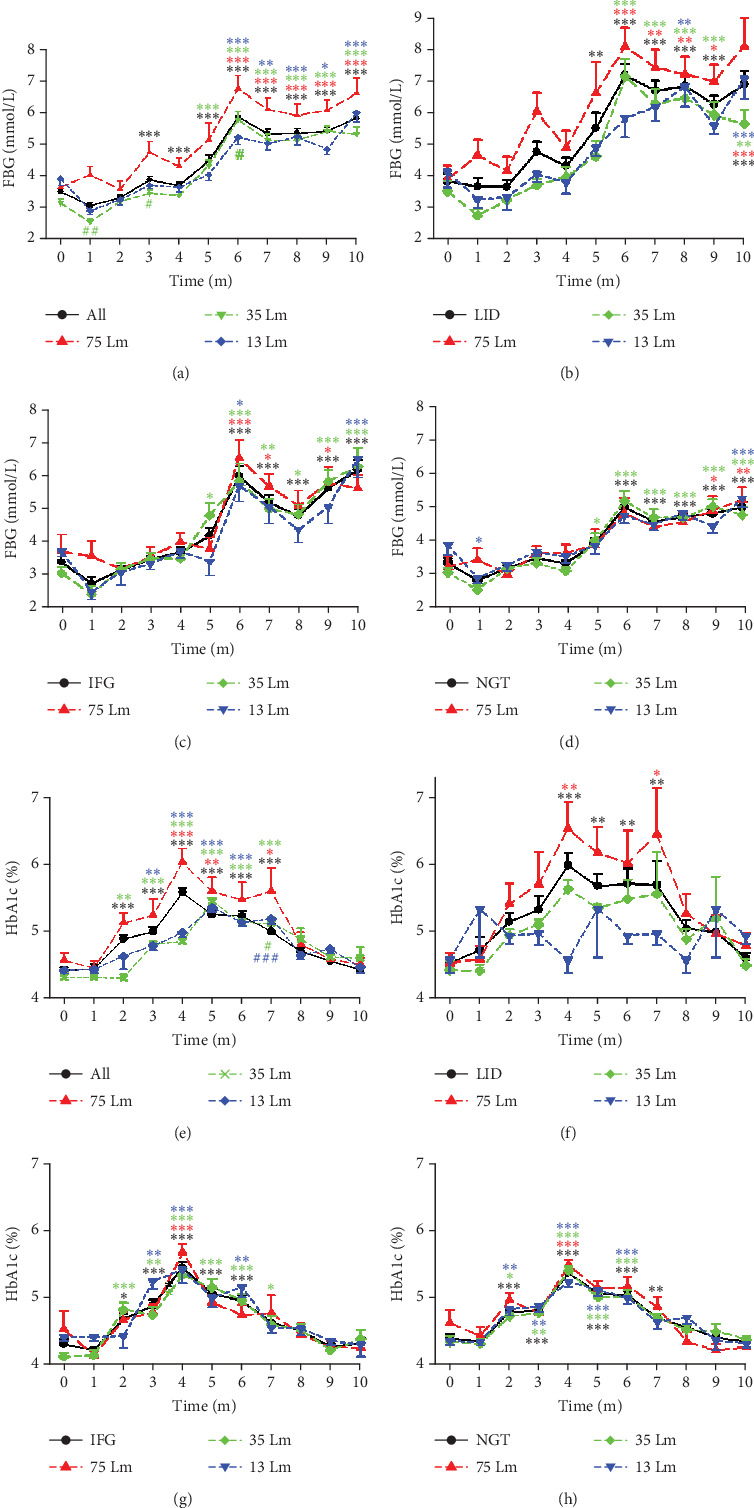
FBG and HbA1c level course in previously healthy monkeys exposed to nocturnal light. Nocturnal light exposure pollution intensity and time-dependently led to (i) (a–d) a consistent increase in FBG levels for the initial 6 months, with these levels persisting at elevated states thereafter and (ii) (e–h) a comparable elevation in HbA1c levels for the first 4 months, followed by a steady decline until ultimately approaching normal levels. Time, experimental time in months (m); 75, 35, and 13 Lm, median nocturnal light intensities of 75, 35, and 13 Lm, respectively; ⁣^∗^*p* < 0.05, ⁣^∗∗^*p* < 0.01, and ⁣^∗∗∗^*p* < 0.001 versus 0 of the same group; #*p* < 0.05, ##*p* < 0.01, and ###*p* < 0.001 versus 75 Lm at the same time point, and distinguished by different colors.

Although monkeys in the LID, IFG, and NGT groups entered the experiment with normal glucose metabolism status, their averaged FBG levels gradually increased during the first 6 months and remained high in the following months (Figures 3(a) and 3(b)). Exposure to nocturnal light elevated the FBG concentration significantly by the third month of the experiment for the entire group, with noticeable differences appearing by the fifth month in the LID group and by the sixth month in both the IFG and NGT groups. Furthermore, the concentration of FBG in monkeys exposed to 75 Lm of nocturnal light remained higher throughout the entire session. There were statistically significant differences in the FBG concentrations between monkeys exposed to 75 and 35 Lm of light at months 1, 3, and 6 (Figure 3(a)). However, the FBG concentrations in the IFG and NGT groups showed no significant difference at any time point under different light intensities. Nevertheless, a similar upward trend was observed in both groups, similar to that seen in the overall and LID groups. Although the study was terminated at month 10, the FBG level in the NGT group appeared to continue increasing up to that month (Figures 3(a), 3(b), 3(c), and 3(d)).

After calculating the morbidities of LID and LID+IFG in monkeys subjected to varying degrees of light pollution, a linear regression analysis revealed a significant positive correlation between the light intensities and the morbidities (Table 2). However, due to the limited number of dependent and independent variables, as well as a *p* value exceeding 0.05, it is possible that this correlation may not be conclusive based on the current sample size.

The point-to-point *t*-test results revealed that the average FBG concentrations of monkeys exposed to 75 Lm were consistently and significantly higher (results not shown). Subsequent linear regression analysis, which utilized median light intensities as independent variables and the average FBG concentration of each monkey living in the corresponding environment as the dependent variable, demonstrated that FBG levels were dependent on light intensity in both the LID and IFG groups (Table 3). However, a month-by-month analysis revealed that the impairment of the light to FBG was not consistent, with the initial month being the most susceptible period (Table 4 and Supporting Information 6).

In each group, there were general similarities between the line arrays representing HbA1c concentrations and those representing FBG. For instance, in the overall and LID groups, the 75 Lm HbA1c lines were above the other lines, albeit with varying peak times. Conversely, in the IFG and NGT groups, the lines were intertwined. The position of the line indicating 75 Lm brightness potentially reinforces the notion of light pollution's intensity-dependent impairing effect; however, linear regression analysis did not reveal any notable disparities in the average HbA1c concentrations or light intensities among the monkey groups (Figures 3(e), 3(f), 3(g), and 3(h)). However, unlike the consistently increasing concentrations of FBG during the initial 6 months, the elevation in HbA1c levels reached its peak at Month 4 and subsequently returned to a level comparable to the baseline. Additionally, when compared to the baseline, the concentrations of HbA1c in the overall, IFG, and NGT groups were significantly elevated starting at Month 2, which was significantly earlier than the Month 6 time point observed for FBG. Considering that HbA1c reflects the average blood glucose levels rather than real-time concentrations, this phenomenon might indicate that, upon exposure to light pollution, the blood glucose concentrations were significantly elevated in the initial months following meals. After further calculation, we found a moderate correlation between the average HbA1c concentration during the Experimental Months 2–5 and light intensity. The correlation coefficients were *R* = 0.275 (*p* = 0.002, *N* = 127) for the overall group and *R* = 0.346 (*P* = 0.033, *N* = 38) for the LID group. These tampered coefficients suggest a reliable correlation between FBG and light pollution in both groups during Months 2–5, but not in the IFG or NGT groups (Figures 3(e), 3(f), 3(g), and 3(h)).

### 3.4. Light Pollution–Induced Diabetes as Reflected by Insulin and C-Peptide Production

**Figure 4:**
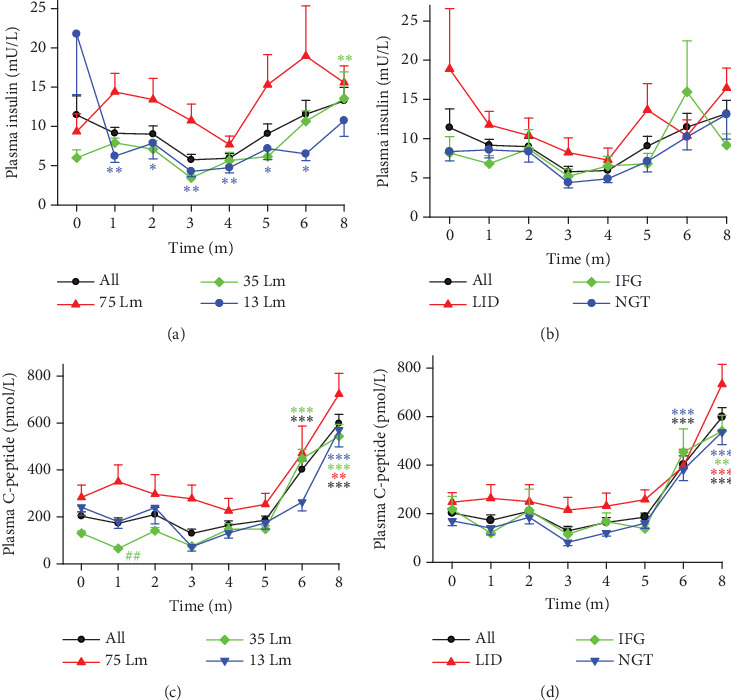
The average concentrations of fasting insulin and C-peptide in monkeys showing the average concentrations of fasting (a, b) insulin and (c, d) C-peptide during Experimental Months 0–8. ⁣^∗^*p* < 0.05, ⁣^∗∗^*p* < 0.01, and ⁣^∗∗∗^*p* < 0.001 versus baseline (0) of the same group; #*p* < 0.05 and ##*p* < 0.01 versus 75 Lm at the same time point.

Insulin is the hormone produced in the pancreas that enables glucose to enter cells [23]. A characteristic of T2D is insulin resistance, in which the body cells cannot functionally use insulin despite a normal or even higher than normal circulating concentration. Studies have shown that insulin sensitivity decreases in situations such as misaligned circadian rhythms or reduced sleep duration [24, 25]. We have now discovered that the production of insulin increased in monkeys that were exposed to the experimental environments. On one hand, the one-way ANOVA and the time course of fasting insulin concentrations revealed the following: (1) There were no significant statistical differences observed between different light intensities (Figure 4(a)) or among various monkey groups (Figure 4(b)). (2) The insulin concentration in the 35 Lm group of monkeys was statistically elevated at Month 8 compared to the baseline (Figure 4(a)). (3) While the insulin production appeared to be suppressed in the 13 Lm group of monkeys, this suppression might have been attributed to an exceptionally high insulin concentration in an individual at the baseline. On the other hand, during the experimental months of 1–8, a Mann–Whitney rank sum test revealed that, when comparing 75–35 Lm or 13 Lm environments, exposure to 75 Lm led to (1) elevated average insulin concentrations in monkey groups across most time points (Figure 4(a)), and (2) the line depicting the average insulin levels for LID monkeys was higher than that of the IFG and NGT groups (Figure 4(b)). Taking into account the rapid clearance rate of insulin (with a half-life of only 5 min), our findings revealed the following: (1) insulin production was not substantially diminished in any of the monkey groups, (2) exposure to brighter nocturnal lighting led to increased insulin resistance, and (3) there was a varying degree of susceptibility to glucose metabolic disorders among the monkeys, with those in the LID group being more susceptible.

The estimation of insulin production can be more precisely determined by measuring plasma C-peptide levels [26]. According to the results obtained from the ELISA test on the plasma of the entire batch of monkeys, the C-peptide levels were statistically elevated at Months 6 and 8 compared to Months 1–5 (Figures 4(c) and 4(d)). This elevation indicates heightened insulin production by the end of Experimental Month 6.

Based on the lines depicting insulin and C-peptide concentrations, it was observed that monkeys exposed to 75 Lm nocturnal light or those in the LID group exhibited higher insulin production compared to monkeys exposed to 35 and 13 Lm, or those in the IFG and NGT groups (Figures 4(c) and 4(d)). When considering light intensity as a standalone factor, a positive correlation was noted between the concentrations of insulin/C-peptide and the light intensities in all monkey groups, including the LID and NGT groups (Table 5). Despite experiencing a higher insulin production following exposure to a light-polluted environment, the FBG levels in monkeys of LID, IFG, and NGT groups increased over the course of the experiment. These findings suggest that, after prolonged exposure to the light-polluted environment, monkeys developed insulin resistance. Consequently, the insulin production was unable to meet the glucose metabolism demands, leading to a disruption in homeostasis and subsequently resulting in IFG and T2D.

### 3.5. The Involvement of Age, Weight, and Sex in the Development of Glucose Metabolic Disorders

**Figure 5:**
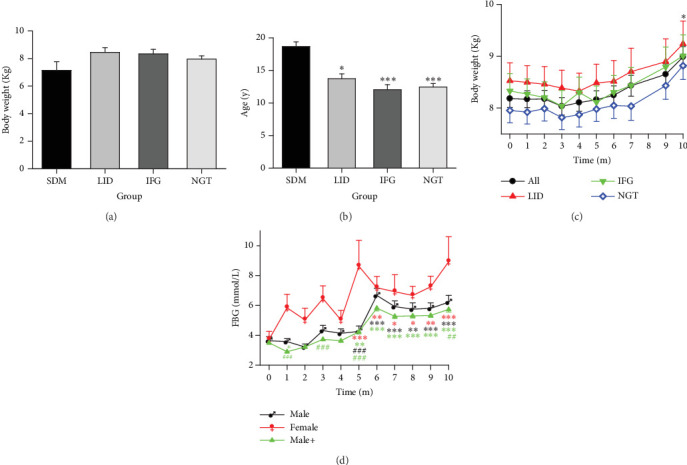
The impact effect of age, weight, and sex in glucose metabolism disorder. (a) Averaged weight in monkey groups showing that no significant statistical differences exist among the groups. (b) The average age of monkey groups showing that age is associated with the occurrence of spontaneous diabetes cases but not with new cases. ⁣^∗^*p* < 0.05 and ⁣^∗∗∗^*p* < 0.001 versus the SDM group. (c) The time course of weight in previously healthy monkeys. ⁣^∗^*p* < 0.05 versus Time Point 0 in overall but not subgroups. (d) FBG concentrations in female and male monkeys. Male, monkeys living in the same environment (75 Lm) with females; Male+, all males in the LID, IFG, and NGT groups. ⁣^∗^*p* < 0.05, ⁣^∗∗^*p* < 0.01, and ⁣^∗∗∗^*p* < 0.001 versus Time Point 0 of the same group; ##*p* < 0.01 and ###*p* < 0.001 versus female at the same time point, and distinguished by different colors.

Although monkeys were not specifically allocated to nocturnal light intensity by age or weight, when all four groups were compared together, the SDM group had statistically older average age, while there was no significant difference among the other three groups (Figure 5(a), repeated one-way ANOVA, *F* = 6.507, *p* = 0.0004, *N* = 137, DF = 136). However, even when comparing group data from previously healthy monkeys with age as an independent variable, linear regression again demonstrated a weak correlation between age and the average FBG concentration (Table 6).

#### 3.5.1. Weight

To ascertain the contribution of weight to the progression of diabetes, a comparison was conducted among the initial weights of four groups, yielding no notable disparities (Figure 5(a), ANOVA, *F* = 1.376, *p* = 0.253, *N* = 137, DF = 136). Moreover, although the average weights of the LID, IFG, and NGT monkey groups demonstrated comparable weight gain trends, no distinct differences were noted within or between the subgroups (Figure 5(b), ANOVA, *F* = 1.362, *p* = 0.096, *N* = 128, DF = 1219). Upon amalgamating the three groups, a statistically significant difference was observed between their initial average weight and their weight at Month 10 (Figure 5(b), ANOVA, *F* = 2.643, *p* = 0.0049, *N* = 128, DF = 1216). This indicates that during the experimental session, the majority of the monkeys experienced an increase in weight, despite three monkeys in the LID group losing weight for over 6 consecutive months and losing 10% or more in total. Regression analysis results showed that, in previously healthy monkeys, there was weak or no correlation between the average weight of each monkey and its averaged FBG (Table 6). This meant that weight alone was not a significant impairing factor in glucose metabolism.

#### 3.5.2. Age

The results depicted in Figure 5(c) indicate that aging, in and of itself, is a contributing factor to spontaneous diabetes. However, it was not a significant impairing factor for newly diagnosed diabetes and IFG cases during the 10-month experimental period. In other words, regardless of age, the glucose metabolism of monkeys exposed to night light might be similarly susceptible to impairment. The results of the regression analysis revealed a moderate correlation between the age of each monkey and its average FBG levels (Table 6), suggesting that aging, as a factor, had a more significant impact on glucose metabolism compared to weight.

#### 3.5.3. Gender

All the female monkeys in this study were housed in rooms with a nocturnal median brightness of 75 Lm. We first compared the monthly tested FBG concentrations between females (*F* = 2.423, *p* = 0.0172, DF = 69, *N* = 7, excluding monkeys in the SDM group) and males living exclusively in this environment (*F* = 12.187, *p* < 0.0001, DF = 280, *N* = 27). One-way ANOVA results indicated that females consistently had higher FBG concentrations, though a statistically significant difference was observed only at Month 5 (p < 0.0001) (Figure 5(d)). Furthermore, when the FBG data from all previously healthy male participants in the study were included (Figure 5(d), male+, *F* = 72.494, *p* < 0.0001, DF = 1287, *N* = 121), the contrast became even more pronounced (*F* = 40.816, *p* < 0.0001, DF = 1357, *N* = 128). As a result, significant differences were observed in Months 1, 3, 5, and 10. These findings suggest that the glucose metabolism in female monkeys is more susceptible to the adverse effects of light pollution.

### 3.6. The Synergistic Effect of Light Pollution, Age, and Weight in the Development of Glucose Metabolic Disorders

**Figure 6:**
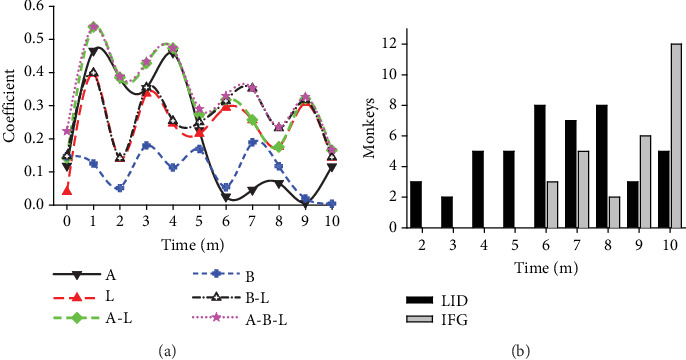
The synergistic impairing effect of light, age, and weight on glucose metabolism. (a) The time course of the correlation coefficient between the influencing factors and the monthly tested FBG concentrations in monkeys categorized as LID, IFG, and NGT groups (*N* = 127). This illustrates the profound impact of sleep environment light (L), age (A), weight (B), or combined effects of age and light (A-L), weight and light (B-L), and age plus weight plus light (A-B-L) on glucose metabolism disability in monkeys. (b) Newly counted LID and IFG cases in the experimental session. Coefficient, correlation coefficient between impact factors and FBG concentrations; time, experimental time in months (m).

As previously mentioned, there was a weak or nonexistent correlation between weight and age with the average FBG level. Interestingly, in the presence of light pollution, multiple linear regressions indicated that both age and weight were significant factors that negatively impacted glucose metabolism in previously healthy monkeys (Table 6). A comparison clearly demonstrated this synergistic effect when examining the time course of the correlation coefficient between the monthly averaged FBG concentrations and factors such as light, age, weight, or any combination in previously healthy animals (Figure 6(a)). In parallelism with Table 6, the coefficients revealed that (1) the impact power of the light-polluted environment on glucose metabolism was uneven throughout the experimental session, with the initial month experiencing the most significant impact. (2) As a single determining factor in glucose metabolism, weight had the least impact, while age was the most powerful, with light in the middle. (3) When combined, age–weight–light had the most powerful impact on glucose metabolism disorders.

The monthly counting revealed that during the first 6 months, half of the LID monkeys (20/40) met the diabetes criteria, while the other half achieved it during Months 7–10. Furthermore, an additional 27 monkeys met the IFG criteria during Months 6–10, as shown in Figure 6(b). These trends suggested that, as regular exposure to the experimental environment was prolonged, the incidence of diabetes and IFG in monkeys increased. However, due to the limited number of animals in the study, the total number of monkeys with glucose metabolism issues was limited, and consequently, the number of healthy monkeys decreased.

## 4. Discussion

A natural circadian rhythm is crucial for maintaining bodily physiological functions, and any unusual factors that disrupt it can adversely affect an individual's health. In our study, we manipulated the light conditions of sleeping rooms for monkeys to mimic the daytime condition of the urban population around the Tropic of Cancer, except for adding light pollution in dark hours. Only the daily circadian was considered in the experiment. All other living factors are maintained constant. Variables such as annual circadian, seasonal changes, weather, and transition points of light on and off were not considered significant factors during analysis. Our findings revealed higher mortality in monkeys with preexisting diabetes and a significant increase in the incidences of diabetes and IFG in previously healthy monkeys. Primates exposed to this environment may experience a reduction in the duration of their nighttime sleep, an increase in the frequency of arousals and awakenings after the onset of sleep, and other disturbances that can usually be avoided during the dark phase of their natural circadian rhythm. Given that the circadian rhythm remained consistent over the 10-month experiment and the sleep environment was otherwise as usual, we believe that light pollution, rather than sleep duration or circadian per se, is a more significant harmful factor in the aggravation or induction of glucose metabolism disorders. The experimental result reinforces the notion that the glucose metabolism conditions in the vast majority of the monkeys were impaired by nocturnal light pollution in an intensity-dependent manner.

In this study, the animals were grouped retrospectively, and data were obtained from tests and samples taken every 4 weeks. Possibly, due to different individual susceptibility or genetic reasons [27], the variations were quite obvious within some groups. The authors consider variation as the likely factor that affected the cyclical model of FBG in Figure 6 and habituation to the light as the reason for the declining trend of FBG and weakened correlations between FBG and light over time. The first peak at Week 4 (Point 1) reflected the sharpest uprush of the FBG. A decline in test FBG would follow once the metabolism system eventually habituated to the living environment.

A light-polluted nocturnal environment may disrupt the production and secretion of melatonin, a hormone produced and secreted by pinealocytes and extrapineal cells during the night. Theoretically, melatonin plays a role in timing circadian rhythms [28] and regulates glucose metabolism [29] through receptor-dependent influences on glucagon and insulin secretion [30–32]. The night light pollution in this study likely depressed the melatonin production, thus inducing glucose metabolism disturbance.

Although both insulin and glucagon are modulated by melatonin, their secretion occurs at different times and rates. In pancreatic islets, the Melatonin Receptor Type 1 (MT1) is expressed on *α*-cells while Type 2 (MT2) is on *β*-cells [30, 32]. Once secreted, melatonin increases glucagon production from *α*-cells immediately [31] but inhibits insulin production in *β*-cells [33] with a functional phase shift [34]. In the case of T2D, insulin secretion may lose a portion of its negative regulatory mechanism, leading to hyperinsulinemia [35]. Moreover, individuals with T2D who experience pineal gland secretion failure will concurrently display hyperglycemia and hyperinsulinemia [36].

Another reason for the differential release of hormones by *α*- and *β*-cells is that acute exposure of MT2 to physiological or supraphysiological concentrations of melatonin induces a concentration- and time-dependent receptor desensitization and internalization, which takes 8–24 h to resensitize [37]. In contrast, prolonged exposure to a melatonin concentration mimicking nocturnal levels does not affect the number of MT1, their affinity, or functional sensitivity [38]. Rather, *α*-cell activation might play a prerequisite role in *β*-cell activation: (i) without the involvement of MT1, melatonin treatment would not yield significant effects on insulin release [37], and (ii) several single nucleotide polymorphisms in the MTNR1B gene, which encodes for MT2, are implicated in the pathogenesis of T2D [39]. Nevertheless, loss-of-function mutations in or removal of MT1 would substantially impair the capacity to metabolize glucose [40].

Over the past few decades, the world has observed a significant surge in the number of individuals diagnosed with diabetes. Despite desperate efforts, attempts to curb this rise have proven ineffective [41, 42]. Our study provides evidence that nocturnal light pollution is directly related to the onset of diabetes and IFG in nonhuman primates, with a dependence on its intensity. It is worth noting that the term “intensity” used here pertains to the level of light pollution rather than the light itself. As an illustration, the constantly changing neon light, even at a median of 13 Lm, might have a pollution efficacy similar to or equivalent to that of the median 75 Lm white light.

**Figure 7:**
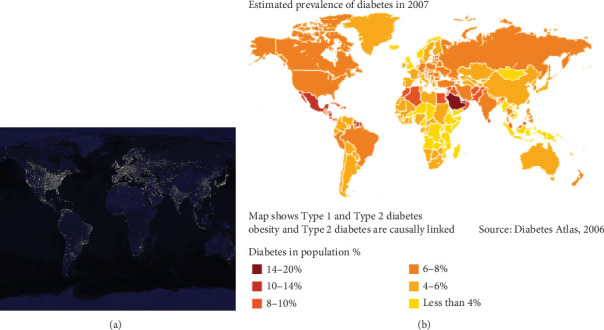
A comparison between a satellite map of Earth at night from (a) NASA (2008) and (b) the world diabetes prevalence map (2007) showing a causal link between the brightness of an area at night and the prevalence of diabetes, where brighter areas tend to have higher diabetes morbidity rates.

Cynomolgus, as primates, are comparable to humans in nearly every aspect, including anatomy, physiology, behavior, sociality, and live habituation, with the exception of the artificial circadian and nocturnal brightness in modern society. Furthermore, humans and monkeys may encounter an identical mechanism impeding glucose metabolism when exposed to nocturnal light for an extended period. The results of the present study could potentially provide valuable insight into the escalating global rates of diabetes and IGT, particularly in industrialized regions. To some extent, a comparison between a satellite map of Earth at night from NASA (2008) [43], which represents the nocturnal brightness of the world and possibly also the urbanization degree of an area, and the world diabetes prevalence map (2007) [44], which shows the prevalence of glucose metabolism disorders, reveals a clearer causal link between the nocturnal brightness and the prevalence of diabetes. This suggests that the brighter an area, the higher the diabetes morbidity (Figure 7). Taking into account the global population size, the rapid pace of urbanization, and the profound impact of light pollution on glucose metabolism, it is possible to explain and anticipate the increased prevalence of diabetes and IGT in humans. Consequently, the appropriate action in this field could be to educate individuals on protecting themselves from the harmful effects of light pollution, thereby addressing the escalating rates of diabetes and IFG. Meanwhile, in our daily lives and urbanization process, it is essential to bear in mind the detrimental effects of light pollution on glucose metabolism and actively take measures to avoid it.

## 5. Conclusion

This research shows that exposure to nocturnal light pollution leads to an increased mortality rate among individuals with preexisting diabetes. In healthy primates, this light pollution has been found to induce elevated incidences of IGT and diabetes. Additionally, light pollution exacerbates glucose metabolism issues in conjunction with aging and weight. In summary, nocturnal light pollution is closely linked to the significant rise in T2D and IGT observed over the past few decades. We also suggest that impaired glucose metabolism may be a secondary consequence of disrupted melatonin secretion caused by night light exposure.

## Figures and Tables

**Table 1:** The diagnostic criteria and number count of monkeys in this study.

**Group**	**Diagnostic criteria**	**N** ^a^	**Rates**
SDM	(1) FBG ≥ 11.1 mmol/L at baseline	4	5.84 (8/137)
	(2) FBG ≥ 7.0 mmol/L and/or HbA1c ≥ 6.5% for consecutive two times starting from baseline	4	

LID	(1) FBG ≥ 11.1 mmol/L once after baseline	5	31.01 (40/129)
	(2) FBG ≥ 7.0 mmol/L nonconsecutive twice	25	
	(3) HbA1c ≥ 6.5% once after baseline	7	
	(4) 11.1 > FBG ≥ 7.0 mmol/L measured once after baseline, with a consistent weight loss for over 6 months, resulting in a final loss of 10% or more	3	

IFG	70mmol/LFBG61mmol/L and/or 6.5 > HbA1c ≥ 6.1%, twice	27	20.93 (27/129)

NGT	The remaining besides SDM, LID, and IFG	62	48.06 (62/129)

*Note:* Listed the diagnostic criteria of spontaneous diabetes mellitus, light-induced diabetes, and impaired fasting glucose, as well as the final number of monkeys in each group.

Abbreviations: FBG, fasting blood glucose; HbA1c, glycosylated hemoglobin; IFG, impaired fasting glucose tolerance; LID, light-induced diabetes; NGT, normal glucose tolerance; SDM, spontaneous diabetes mellitus.

^a^Based on the first reached criteria.

**Table 2:** The correlation between the rate of glucose metabolic disorder and light intensity in previously healthy monkeys.

	**75 Lm**	**35 Lm**	**13 Lm**	**Correlation**			
				**Formula**	**R**	**F**	**p** ** value**
LID	48.57% (17/35)	22.81% (13/57)	27.03% (10/37)	*Y* = 17.082 + 0.383 *L*	0.872	3.183	0.325
IFG	20% (7/35)	22.81% (13/57)	18.92% (7/37)	*Y* = 20.299 + 0.007 *L*	0.106	0.0114	0.932
LID+IFG	68.57% (24/35)	45.61% (26/57)	45.95% (17/37)	*Y* = 37.377 + 0.39 *L*	0.932	6.63	0.236
NGT	31.43% (11/35)	54.39% (31/57)	54.05% (20/37)	*Y* = 62.623 − 0.39 *L*	−0.932	6.63	0.236

*Note:R*, the coefficient of correlation; *Y*, the predicted glucose metabolic morbidity in response to *L*.

Abbreviations: *F*, *F* value; IFG, impaired fasting glucose tolerance; *L*, light intensity; LID, light-induced diabetes; NGT, normal glucose tolerance.

**Table 3:** The correlation between FBG and light pollution in previously healthy monkeys.

	**Formula**	**R**	**F**	**p** ** value**
All (128)	*G* = 3.957 + 0.016 *L*	0.371	20.172	< 0.001
LID (39)	*G* = 4.32 + 0.025 *L*	0.498	12.223	< 0.001
IFG (27)	*G* = 4.252 + 0.0062 *L*	0.407	4.975	0.035
NGT (62)	*G* = 4.038 + 0.000801 *L*	0.0361	0.0784	0.78

*Note:* All, whole batch of monkeys; FBG, concentration in response to light intensity; *G*, the predicted FBG concentration; *R*, the coefficient of correlation.

Abbreviations: *F*, *F* value; IFG, impaired fasting glucose tolerance; *L*, light intensity; LID, light-induced diabetes; NGT, normal glucose tolerance.

**Table 4:** The month-by-month correlation of FBG with light, age, weight, or their combined effects.

**Month**	**FGB-light, G(FGB) =**	**R**	**F**	**p** ** value**
1 (128)	2.255 + 0.0206 *L*	0.397	23.509	< 0.001
3 (127)	3.126 + 0.0191 *L*	0.337	15.963	< 0.001
4 (126)	3.214 + 0.0126 *L*	0.246	7.992	0.005
5 (125)	3.752 + 0.0183 *L*	0.215	5.964	0.016
6 (120)	4.931 + 0.0246 *L*	0.294	11.151	0.001
7 (121)	4.646 + 0.0183 *L*	0.256	8.329	0.005
9 (121)	4.679 + 0.0195 *L*	0.308	12.451	< 0.001

**Month**	**FBG-age, G=**	**R**	**F**	**p** ** value**
1 (128)	1.4 + 0.13*A*	0.466	34.934	< 0.001
2 (127)	2.179 + 0.0893*A*	0.384	21.6	< 0.001
3 (127)	2.506 + 0.108*A*	0.353	17.771	< 0.001
4 (126)	2.101 + 0.126*A*	0.46	32.2	< 0.001
5 (125)	3.166 + 0.103*A*	0.225	6.581	0.012

**Month**	**FBG-age-light, G=**	**R**	**F**	**p** ** value**
1 (128)	1.126 + 0.107*A* + 0.0147 *L*	0.538	25.465	< 0.001
2 (127)	2.156 + 0.0847*A* + 0.00119 *L*	0.385	10.722	< 0.001
3 (127)	2.23 + 0.0845*A* + 0.0144 *L*	0.428	13.928	< 0.001
4 (126)	1.978 + 0.116*A* + 0.00626 *L*	0.474	17.835	< 0.001
5 (125)	2.894 + 0.0809*A* + 0.0139 *L*	0.274	4.945	0.009
6 (120)	5.51 − 0.0556*A* + 0.0279 *L*	0.318	6.572	0.002
7 (121)	04.743 − 0.00921*A* + 0.0188 *L*	0.257	4.167	0.018
9 (121)	5.01 − 0.0314*A* + 0.0213 *L*	0.321	6.771	0.002

**Month**	**FBG-weight**	**R**	**F**	**p** ** value**
3 (127)	4.904 − 0.124*B*	0.18	4.183	0.043
7 (121)	4.019 + 0.161*B*	0.189	4.408	0.038

**Month**	**FBG-weight-light, G=**	**R**	**F**	**p** ** value**
1 (128)	2.510 − 0.0286*B* + 0.0202 *L*	0.399	11.833	< 0.001
3 (127)	3.842 − 0.0805*B* + 0.0178 *L*	0.355	8.961	< 0.001
4 (126)	3.573 − 0.0402*B* + 0.0119 *L*	0.254	4.245	0.016
5 (125)	4.953 − 0.134*B* + 0.016 *L*	0.25	4.062	0.02
6 (120)	3.946 + 0.111*B* + 0.0264 *L*	0.314	6.384	0.002
7 (121)	2.773 + 0.210*B* + 0.0217 *L*	0.352	8.368	< 0.001
8 (121)	3.765 + 0.128*B* + 0.0141 *L*	0.232	3.357	0.038
9 (121)	4.134 + 0.0611*B* + 0.0205 *L*	0.318	6.636	0.002

**Month**	**FBG-age-weight-light, G=**	**R**	**F**	**p** ** value**
1 (128)	0.951 + 0.108*A* + 0.0178*B* + 0.0149 *L*	0.54	16.901	< 0.001
2 (127)	1.91 + 0.0897*A* + 0.0259*B* + 0.0015 *L*	0.39	7.25	< 0.001
3 (127)	2.683 + 0.0805*A* − 0.0461*B* + 0.0139 *L*	0.433	9.465	< 0.001
4 (126)	1.873 + 0.117*A* + 0.0105*B* + 0.00639 *L*	0.474	11.811	< 0.001
5 (125)	3.918 + 0.0719*A* − 0.104*B* + 0.0126 *L*	0.29	3.713	0.013
6 (120)	4.618 − 0.0479*A* + 0.0913*B* + 0.0289 *L*	0.33	4.725	0.004
7 (121)	2.661 + 0.00799*A* + 0.213*B* + 0.0213 *L*	0.353	5.552	0.001
9 (121)	4.518 − 0.0274*A* + 0.0503*B* + 0.0219 *L*	0.327	4.68	0.004

Note: Linear regression analysis results show that the FBG concentrations could be affected by age, weight, light, and their combination. Lines with *p* > 0.05 have been removed; refer to Supporting Information 6 for the complete table. *B*, weight; FBG, concentration in response to light intensity; *G*, the predicted FBG concentration; *R*, the coefficient of correlation.

Abbreviations: *A*, age; *F*, *F* value; *L*, light.

**Table 5:** The correlation between the average fasting insulin/C-peptide concentrations in plasma and light pollution.

**Group**	**Formula**	**R**	**F**	**p** ** value**	**DF**
All (127)	*I* = 3.867 + 0.125 *L*	0.389	22.311	< 0.001	126
LID (38)	*I* = 4.139 + 0.137 *L*	0.426	7.962	0.008	37
IFG (27)	*I* = 3.671 + 0.118 *L*	0.343	3.335	0.08	26
NGT (62)	*I* = 4.198 + 0.108 *L*	0.336	7.648	0.008	61
All (127)	*C* = 166.744 + 2.419 *L*	0.284	10.958	0.001	126
LID (38)	*C* = 181.787 + 3.213 *L*	0.334	4.523	0.04	37
IFG (27)	*C* = 262.224 − 0.37 *L*	0.0457	0.0523	0.821	26
NGT (62)	*C* = 143.7 + 2.354 *L*	0.313	6.497	0.013	61

*Note:* Linear regression analysis results showing the predicted fasting insulin/C-peptide concentrations in response to light. All, whole batch of monkeys; *R*, the coefficient of correlation.

Abbreviations: *C*, C-peptide; DF, degree of freedom; *F*, *F* value; *I*, insulin; IFG, impaired fasting glucose tolerance; *L*, light intensity; LID, light-induced diabetes; NGT, normal glucose tolerance.

**Table 6:** The correlation between the averaged FBG and age, weight, light pollution, or a combination of these factors as examined in different brightness intensities or in previously healthy monkeys.

**Group**	**Formula, G(FGB)=**	**R**	**F**	**p** ** value**
75 Lm (34)	4.358 + 0.0577*A*	0.164	0.888	0. 353
	5.61 − 0.049*B*	0.071	0.161	0. 69

35 Lm (57)	4.132 + 0.0224*A*	0.117	0.763	0. 386
	3.667 + 0.0826*B*	0.274	4.47	0. 039

13 Lm (37)	3.859 + 0.0342*A*	0.216	1.705	< 0.001
	3.705 + 0.0709*B*	0.159	0.902	0.349

All (128)	3.957 + 0.016 *L*	0.371	20.172	< 0.001
	3.734 + 0.0666*A*	0.288	11.398	< 0.001
	4.69 − 0.0129*B*	0.025	0.0764	0.783
	3.482 + 0.0449*A* + 0.0135 *L*	0.415	13.006	< 0.001
	3.703 + 0.0285*B* + 0.0164 *L*	0.375	10.244	< 0.001
	2.995 + 0.0492*A* + 0.0495*B* + 0.0141 *L*	0.425	9.103	< 0.001

LID (39)	4.32 + 0.025 *L*	0.498	12.223	< 0.001
	4.659 + 0.0587*A*	0.206	1.642	0.208
	6.469 − 0.121*B*	0.201	1.565	0.219
	4.294 + 0.00252*A* + 0.0249 *L*	0.498	5.949	0. 006
	4.236 + 0.00827*B* + 0.0253 *L*	0.498	5.951	0.006
	4.181 + 0.00358*A* + 0.01*B* + 0.0251 *L*	0.499	3.86	0.017

IFG (27)	4.252 + 0.0062 *L*	0.407	4.975	0.035
	4.139 + 0.0299*A*	0.343	3.335	0.08
	4.628 − 0.0155*B*	0.072	0.13	0.722
	4.093 + 0.0177*A* + 0.00484 *L*	0.446	2.978	0.07
	4.345 − 0.00616*B* + 0.0108 *L*	0.41	2.423	0.11
	4.127 + 0.017*A* − 0.0036*B* + 0.00485 *L*	0.446	1.906	0.157

NGT (62)	4.038 + 0.000801 *L*	0.036	0.0784	0.78
	3.452 + 0.0493*A*	0.451	15.331	< 0.001
	4.077 − 0.00143*B*	0.006	0.0019	0.965
	3.463 + 0.0496*A* − 0.000431 *L*	0.452	7.555	< 0.001
	4.039 − 0.000144*B* + 0.000799 *L*	0.036	0.0385	0.962
	3.244 + 0.0516*A* + 0.0233*B* − 0.0002 *L*	0.46	5.199	0.003

*Note:* Linear regression analysis results showing the predicted FBG concentration derived from factors of age, weight, light, and any combination of them. All, whole batch of monkeys; *B*, weight; FBG, concentration in response to light intensity; *G*, the predicted FBG concentration; *R*, the coefficient of correlation.

Abbreviations: *A*, age; *F*, *F* value; *L*, light intensity; LID, light-induced diabetes; NGT, normal glucose tolerance.

## Data Availability

The datasets generated during and/or analyzed during the current study are available from the corresponding author on reasonable request.

## Nomenclature

2hGTT: 2 h glucose tolerance test
All: monkeys of the whole patch
FBG: fasting blood glucose
HbA1c: glycosylated hemoglobin
IFG: impaired fasting glucose tolerance
IGT: impaired glucose tolerance
LID: light-induced diabetes
MT1: Melatonin Receptor Type 1
MT2: Melatonin Receptor Type 2
NGT: normal glucose tolerance
SDM: spontaneous diabetes mellitus
T2D: Type 2 diabetes mellitus

## Supporting Information

Supporting Information 1 Table S1. Lighting condition and distribution of monkeys showing the light brightness in experimental rooms day and night and the animal numbers in each lighting condition. F, female; M, male; N, animal number.

Supporting Information 2 Table S2. One-way ANOVA results of FBG in monkeys showing the statistical results of *p* value, *F* value, and degree of freedom within and between groups for FGB in monkeys grouped with light brightness or glucose metabolic condition. All, whole batch of monkeys; DF, degree of freedom; *F*, *F* value; IFG, impaired fasting glucose tolerance; LID, light-induced diabetes; NGT, normal glucose tolerance.

Supporting Information 3 Table S3. One-way ANOVA results of HbA1c in monkeys showing the statistical results of *p* value, *F* value, and degree of freedom within and between groups for HbA1c in monkeys grouped with light brightness or glucose metabolic condition. All, whole batch of monkeys; DF, degree of freedom; *F*, *F* value; IFG, impaired fasting glucose tolerance; LID, light-induced diabetes; NGT, normal glucose tolerance.

Supporting Information 4 Table S4. One-way ANOVA results of insulin in monkeys showing the statistical results of *p* value, *F* value, and degree of freedom within and between groups for insulin in monkeys grouped with light brightness or glucose metabolic condition. All, whole batch of monkeys; DF, degree of freedom; *F*, *F* value; IFG, impaired fasting glucose tolerance; LID, light-induced diabetes; NGT, normal glucose tolerance.

Supporting Information 5 Table S5. One-way ANOVA results of C-peptide in monkeys showing the statistical results of *p* value, *F* value, and degree of freedom within and between groups for C-peptide in monkeys grouped with light brightness or glucose metabolic condition. All, whole batch of monkeys; DF, degree of freedom; *F*, *F* value; IFG, impaired fasting glucose tolerance; LID, light-induced diabetes; NGT, normal glucose tolerance.

Supporting Information 6 Table S6. Month-by-month correlation of FBG with light, age, weight, or their combined effects. Linear regression analysis results showing that the FBG concentrations could be affected by age, weight, light, and their combination, but not evenly as tested month by month. A, age; B, weight; *F*, *F* value; FBG, concentration in response to light intensity; G, the predicted FBG concentration; L, light; R, the coefficient of correlation. Lines with *p* > 0.05 have been removed; refer to Supporting Information 6 for the complete table.
