# Corrigendum to “Nocturnal Light Pollution Synergistically Impairs Glucose Metabolism With Age and Weight in Monkeys”

**DOI:** 10.1155/jdr/9841020

**Published:** 2025-05-28

S. Wang, X. Cheng, Z. Liang, Z. Chen, J. Zhang, Q. Xu, “Nocturnal Light Pollution Synergistically Impairs Glucose Metabolism With Age and Weight in Monkeys,” *Journal of Diabetes Research* 2024, no. 1 (2024): 1–16, https://doi.org/10.1155/2024/5112055

In the article titled “Nocturnal Light Pollution Synergistically Impairs Glucose Metabolism With Age and Weight in Monkeys,” author Jiankai Zhang was affiliated to Department of Anatomy, Guangdong University, Dongguan, China, which is incorrect. The correct affiliation for this author is:

Department of Human Anatomy, Guangdong Medical University, Dongguan, China

We apologize for this error.

