# Supplementary material for: Nocturnal Light Pollution Synergistically Impairs Glucose Metabolism With Age and Weight in Monkeys
Source: J Diabetes Res. 2024 Dec 10;2024:5112055. doi: 10.1155/2024/5112055 (PMC11824604; doi:10.1155/2024/5112055)
Supplement: Supporting Information 1 — Table S1. Lighting condition and distribution of monkeys showing the light brightness in experimental rooms day and night and the animal numbers in each lighting condition. F, female; M, male; N, animal number. [file 5112055.f1.docx]

**Supplementary Table 1. Lighting condition and distribution of monkeys.**

| **Brightness (Lm)** | | | **N/Sex** |
| --- | --- | --- | --- |
| **Daytime** | **Nocturnal (median)** | **Light spectrum** |  |
| 40-200 | 51-100 white (75) | 400-780nm | 10F, 27M |
| 40-200 | 21-50 light-blue (35) | 450nm | 57M |
| 40-200 | 6-20 flashing neon (13) | 400-780nm | 43M |
| Total | | | 137 (10F, 127M) |
