# Supplementary material for: Nocturnal Light Pollution Synergistically Impairs Glucose Metabolism With Age and Weight in Monkeys
Source: J Diabetes Res. 2024 Dec 10;2024:5112055. doi: 10.1155/2024/5112055 (PMC11824604; doi:10.1155/2024/5112055)
Supplement: Supporting Information 3 — Table S3. One-way ANOVA results of HbA1c in monkeys showing the statistical results of p value, F value, and degree of freedom within and between groups for HbA1c in monkeys grouped with light brightness or glucose metabolic condition. All, whole batch of monkeys; DF, degree of freedom; F, F value; IFG, impaired fasting glucose tolerance; LID, light-induced diabetes; NGT, normal glucose tolerance. [file 5112055.f3.docx]

**Supplementary Table 3. One-way ANOVA results of HbA1c in monkeys.**

|  | | **P-value** | **F** | **DF** | |
| --- | --- | --- | --- | --- | --- |
|  |  |  |  | **Between groups** | **Within groups** |
| All (128) | | <0.0001 | 30.316 | 10 | 1346 |
| 75 Lm (34) | | <0.0001 | 7.385 | 10 | 339 |
| 35 Lm (57) | | <0.0001 | 18.153 | 10 | 606 |
| 13 Lm (37) | | <0.0001 | 12.59 | 10 | 378 |
| LID (39) | LID | <0.0001 | 6.304 | 10 | 397 |
|  | 75 Lm (16) | 0.0002 | 3.749 | 10 | 153 |
|  | 35 Lm (13) | 0.0129 | 2.371 | 10 | 132 |
|  | 13 Lm (10) | 0.0643 | 1.842 | 10 | 90 |
| IFG (27) | IFG | <0.0001 | 27.595 | 10 | 285 |
|  | 75 Lm (7) | <0.0001 | 7.168 | 10 | 76 |
|  | 35 Lm (13) | <0.0001 | 15.822 | 10 | 131 |
|  | 13 Lm (7) | <0.0001 | 9.581 | 10 | 66 |
| NGT (62) | NGT | <0.0001 | 40.097 | 10 | 642 |
|  | 75 Lm (11) | <0.0001 | 9.871 | 10 | 99 |
|  | 35 Lm (31) | <0.0001 | 18.801 | 10 | 321 |
|  | 13 Lm (20) | <0.0001 | 14.077 | 10 | 200 |
