# Supplementary material for: Nocturnal Light Pollution Synergistically Impairs Glucose Metabolism With Age and Weight in Monkeys
Source: J Diabetes Res. 2024 Dec 10;2024:5112055. doi: 10.1155/2024/5112055 (PMC11824604; doi:10.1155/2024/5112055)
Supplement: Supporting Information 4 — Table S4. One-way ANOVA results of insulin in monkeys showing the statistical results of p value, F value, and degree of freedom within and between groups for insulin in monkeys grouped with light brightness or glucose metabolic condition. All, whole batch of monkeys; DF, degree of freedom; F, F value; IFG, impaired fasting glucose tolerance; LID, light-induced diabetes; NGT, normal glucose tolerance. [file 5112055.f4.docx]

**Supplementary Table 4. One-way ANOVA results of insulin in monkeys.**

|  | **P-value** | **F** | **DF** | |
| --- | --- | --- | --- | --- |
|  |  |  | **Between groups** | **Within groups** |
| All (127) | 0.0009 | 3.541 | 7 | 969 |
| 75 Lm (34) | 0.2192 | 1.368 | 7 | 261 |
| 35 Lm (57) | <0.0001 | 4.772 | 7 | 428 |
| 13 Lm (36) | 0.0015 | 3.453 | 7 | 271 |
| LID (38) | 0.2171 | 1.372 | 7 | 287 |
| IFG (27) | 0.188 | 1.447 | 7 | 205 |
| NGT (62) | 0.0016 | 3.376 | 7 | 461 |
