# Supplementary material for: Nocturnal Light Pollution Synergistically Impairs Glucose Metabolism With Age and Weight in Monkeys
Source: J Diabetes Res. 2024 Dec 10;2024:5112055. doi: 10.1155/2024/5112055 (PMC11824604; doi:10.1155/2024/5112055)
Supplement: Supporting Information 5 — Table S5. One-way ANOVA results of C-peptide in monkeys showing the statistical results of p value, F value, and degree of freedom within and between groups for C-peptide in monkeys grouped with light brightness or glucose metabolic condition. All, whole batch of monkeys; DF, degree of freedom; F, F value; IFG, impaired fasting glucose tolerance; LID, light-induced diabetes; NGT, normal glucose tolerance. [file 5112055.f5.docx]

**Supplementary Table 5. One-way ANOVA results of C-Peptide in monkeys.**

|  | **P-value** | **F** | **DF** | |
| --- | --- | --- | --- | --- |
|  |  |  | **Between groups** | **Within groups** |
| All (127) | <0.0001 | 35.73 | 7 | 974 |
| 75 Lm (34) | <0.0001 | 4.813 | 7 | 254 |
| 35 Lm (57) | <0.0001 | 49.521 | 7 | 432 |
| 13 Lm (36) | <0.0001 | 12.02 | 7 | 272 |
| LID (38) | <0.0001 | 8.47 | 7 | 287 |
| IFG (27) | <0.0001 | 7.55 | 7 | 206 |
| NGT (62) | <0.0001 | 26.014 | 7 | 465 |
