# Supplementary material for: Nocturnal Light Pollution Synergistically Impairs Glucose Metabolism With Age and Weight in Monkeys
Source: J Diabetes Res. 2024 Dec 10;2024:5112055. doi: 10.1155/2024/5112055 (PMC11824604; doi:10.1155/2024/5112055)
Supplement: Supporting Information 6 — Table S6. Month-by-month correlation of FBG with light, age, weight, or their combined effects. Linear regression analysis results showing that the FBG concentrations could be affected by age, weight, light, and their combination, but not evenly as tested month by month. A, age; B, weight; F, F value; FBG, concentration in response to light intensity; G, the predicted FBG concentration; L, light; R, the coefficient of correlation. Lines with p > 0.05 have been removed; refer to Supporting Information 6 for the complete table. [file 5112055.f6.docx]

**Supplementary Table 6. Month-by-month correlation of FBG with light, age, weight, or their combined effects.**

| **Month** | **Formula (G =)** | **R** | **F** | **P-value** |
| --- | --- | --- | --- | --- |
| 0 (128) | 3.590-0.00197L | 0.039 | 0.196 | 0.659 |
| 1 (128) | 2.255+0.0206L | 0.397 | 23.509 | <0.001 |
| 2 (127) | 3.083+0.00602L | 0.139 | 2.458 | 0.119 |
| 3 (127) | 3.126+0.0191L | 0.337 | 15.963 | <0.001 |
| 4 (126) | 3.214+0.0126L | 0.246 | 7.992 | 0.005 |
| 5 (125) | 3.752+0.0183L | 0.215 | 5.964 | 0.016 |
| 6 (120) | 4.931+0.0246L | 0.294 | 11.151 | 0.001 |
| 7 (121) | 4.646+0.0183L | 0.256 | 8.329 | 0.005 |
| 8 (121) | 4.904+0.0121L | 0.175 | 3.74 | 0.055 |
| 9 (121) | 4.679+0.0195L | 0.308 | 12.451 | <0.001 |
| 10 (114) | 5.387+0.0121L | 0.143 | 2.35 | 0.128 |
| **Month** | **FBG-age, Formula** | **R** | **F** | **P-value** |
| 0 (128) | 3.107+0.0318A | 0.118 | 1.776 | 0.185 |
| 1 (128) | 1.4+0.13A | 0.466 | 34.934 | <0.001 |
| 2 (127) | 2.179+0.0893A | 0.384 | 21.6 | <0.001 |
| 3 (127) | 2.506 +0.108A | 0.353 | 17.771 | <0.001 |
| 4 (126) | 2.101+0.126A | 0.46 | 32.2 | <0.001 |
| 5 (125) | 3.166+0.103A | 0.225 | 6.581 | 0.012 |
| 6 (120) | 6.022+0.0107A | 0.025 | 0.0712 | 0.79 |
| 7 (121) | 0.594+0.0203A | 0.054 | 0.349 | 0.556 |
| 8 (121) | 5.067+0.0238A | 0.066 | 0.517 | 0.474 |
| 9 (121) | 5.407+0.00182A | 0.006 | 0.0036 | 0.952 |
| 10 (114) | 5.181+0.0534A | 0.116 | 1.528 | 0.219 |
| **Month** | **FBG-age-light, Formula** | **R** | **F** | **P-value** |
| 0 (128) | 3.184+0.0384A-0.00412L | 0.142 | 1.278 | 0.282 |
| 1 (128) | 1.126+0.107A+0.0147L | 0.538 | 25.465 | <0.001 |
| 2 (127) | 2.156+0.0847A+0.00119L | 0.385 | 10.722 | <0.001 |
| 3 (127) | 2.23+0.0845A+0.0144L | 0.428 | 13.928 | <0.001 |
| 4 (126) | 1.978+0.116A+0.00626L | 0.474 | 17.835 | <0.001 |
| 5 (125) | 2.894+0.0809A+0.0139L | 0.274 | 4.945 | 0.009 |
| 6 (120) | 5.51-0.0556A+0.0279L | 0.318 | 6.572 | 0.002 |
| 7 (121) | 04.743-0.00921A+0.0188L | 0.257 | 4.167 | 0.018 |
| 8 (121) | 4.847+0.00538A+0.0118L | 0.175 | 1.867 | 0.159 |
| 9 (121) | 5.01-0.0314A+0.0213L | 0.321 | 6.771 | 0.002 |
| 10 (114) | 4.979+0.0386A+0.0102L | 0.165 | 1.545 | 0.218 |
| **Month** | **FBG-body weight, Formula** | **R** | **F** | **P-value** |
| 0 (128) | 2.771+0.0899B | 0.147 | 2.789 | 0.097 |
| 1 (128) | 3.719-0.793B | 0.125 | 1.998 | 0.16 |
| 2 (127) | 3.54-0.0267B | 0.051 | 0.321 | 0.572 |
| 3 (127) | 4.904-0.124B | 0.18 | 4.183 | 0.043 |
| 4 (126) | 4.295-0.0703B | 0.113 | 1.61 | 0.207 |
| 5 (125) | 5.924-0.175B | 0.169 | 3.637 | 0.059 |
| 6 (120) | 5.447+0.053B | 0.053 | 0.337 | 0.562 |
| 7 (121) | 4.019+0.161B | 0.189 | 4.408 | 0.038 |
| 8 (121) | 4.576+0.0956B | 0.117 | 1.639 | 0.203 |
| 9 (121) | 5.309+0.0146B | 0.019 | 0.0449 | 0.832 |
| 10 (114) | 5.875-0.00361B | 0.004 | 0.0014 | 0.971 |
| **Month** | **FBG-body weight-light, Formula** | **R** | **F** | **P-value** |
| 0 (128) | 2.799+0.0887B-0.000472L | 0.147 | 1.389 | 0.253 |
| 1 (128) | 2.510-0.0286B+0.0202L | 0.399 | 11.833 | <0.001 |
| 2 (127) | 3.193-0.0124B+0.00582L | 0.141 | 1.253 | 0.289 |
| 3 (127) | 3.842-0.0805B+0.0178L | 0.355 | 8.961 | <0.001 |
| 4 (126) | 3.573-0.0402B+0.0119L | 0.254 | 4.245 | 0.016 |
| 5 (125) | 4.953-0.134B+0.016L | 0.25 | 4.062 | 0.02 |
| 6 (120) | 3.946+0.111B+0.0264L | 0.314 | 6.384 | 0.002 |
| 7 (121) | 2.773+0.210B+0.0217L | 0.352 | 8.368 | <0.001 |
| 8 (121) | 3.765+0.128B+0.0141L | 0.232 | 3.357 | 0.038 |
| 9 (121) | 4.134+0.0611B+0.0205L | 0.318 | 6.636 | 0.002 |
| 10 (114) | 5.254+0.0151B+0.0122L | 0.144 | 1.177 | 0.312 |
| **Month** | **FBG-age-body weight-light, Formula** | **R** | **F** | **P-value** |
| 0 (128) | 2.108+0.048A+0.109B-0.0028L | 0.223 | 2.154 | 0.097 |
| 1 (128) | 0.951+0.108A+0.0178B+0.0149L | 0.54 | 16.901 | <0.001 |
| 2 (127) | 1.91+0.0897A+0.0259B+0.0015L | 0.39 | 7.25 | <0.001 |
| 3 (127) | 2.683+0.0805A-0.0461B+0.0139L | 0.433 | 9.465 | <0.001 |
| 4 (126) | 1.873+0.117A+0.0105B+0.00639L | 0.474 | 11.811 | <0.001 |
| 5 (125) | 3.918+0.0719A-0.104B+0.0126L | 0.29 | 3.713 | 0.013 |
| 6 (120) | 4.618-0.0479A+0.0913B+0.0289L | 0.33 | 4.725 | 0.004 |
| 7 (121) | 2.661+0.00799A+0.213B+0.0213L | 0.353 | 5.552 | 0.001 |
| 8 (121) | 3.537+0.0162A+0.134B+0.0133L | 0.236 | 2.296 | 0.081 |
| 9 (121) | 4.518-0.0274A+0.0503B+0.0219L | 0.327 | 4.68 | 0.004 |
| 10 (114) | 4.754+0.0398A+0.0242B+0.0104L | 0.166 | 1.041 | 0.377 |
